# Evaluating immune responses to pneumococcal vaccines

**DOI:** 10.5415/apallergy.0000000000000114

**Published:** 2023-09-07

**Authors:** Bernard Yu-Hor Thong, Ruby Pawankar, Hae-Sim Park, Amir Hamzah Abdul Latiff

**Affiliations:** 1 Department of Rheumatology, Allergy and Immunology, Tan Tock Seng Hospital, Singapore; 2 Department of Pediatrics, Nippon Medical School, Tokyo, Japan; 3 Department of Allergy and Clinical Immunology, Ajou University School of Medicine, Suwon, South Korea; 4 Department of Biomedical Sciences, Ajou University School of Medicine, Suwon, South Korea; 5 Allergy & Immunology Centre Pantai Hospital Kuala Lumpur, Malaysia; 6 Sunway Centre for Planetary Health, Sunway University, Petaling Jaya, Malaysia

**Keywords:** Adverse effects, immunization schedule, pneumococcal vaccines, vaccine efficacy

## Abstract

*Streptococcus pneumoniae* (pneumococcus) is a significant cause of bacterial infections ranging from mild infections affecting the respiratory tract such as otitis media and sinusitis to severe diseases including bacteremia, pneumonia, and invasive pneumococcal disease (IPD) (eg, meningitis, septic arthritis, and endocarditis). Pneumococcal vaccines were first developed in the 1970s as capsular pneumococcal polysaccharide vaccines, which were T-cell independent and hence lacked immunologic memory. Subsequently in the year 2000, pneumococcal conjugate vaccines (PCV) conjugated to a protein to increase immunogenicity were developed and made commercially available. The increasing number of pneumococcal serotypes identified and the expanding pipeline of PCV vaccines with improved immunogenicity have significantly reduced the morbidity and mortality associated with IPD in high-risk patients. Pneumococcal vaccines also play an important role in the diagnosis and immunophenotyping of children and adults with inborn errors of immunity (IEI) given the increasing diversity/heterogeneity of IEI presenting with primary and/or specific antibody deficiency. Other than the quantitation of serotype levels in routine clinical care, other measurements of immune response including the functional activity of antibodies, antibody avidity, cell-mediated immunity, and immunological memory remain limited to clinical trials during vaccine development.

*Streptococcus pneumoniae* (*S. pneumoniae*, pneumococcus) is a significant cause of bacterial infections ranging from mild infections affecting the respiratory tract for example otitis media, sinusitis, and bronchitis to more severe diseases for example bacteremia, pneumonia, and invasive pneumococcal disease (IPD) (eg, meningitis and septic arthritis) [1]. Most cases occur in children under 5 years of age and the elderly. The epidemiology of pneumococcal disease varies by geographical region, with the highest incidence and mortality rates across Southeast Asia and Africa [2, 3]. *S. pneumoniae* was first isolated by Louis Pasteur in 1881 from the saliva of a patient with rabies. It is a Gram-positive diplococcus, facultative anaerobic organism with 100 serotypes documented as of 2020. Serotypes are of varying virulence with only a few causing most pneumococcal infections. The association of different serotypes with disease severity (IPD vs non-IPD) and antimicrobial resistance in different age groups and geographical regions may be modified by national vaccination programs and pneumococcal conjugated vaccine (PCV) coverage [4].

## 1. Pathogenesis of pneumococcal disease

Pneumococcal colonization precedes pneumococcal disease [5]. The human nasopharynx is the only reservoir of pneumococci, which are transmitted by droplet spread between individuals. Asymptomatic carriage varies from 20% to 60% in school-age children, to 5% to 10% in adults. A systematic review has shown that factors positively associated with pneumococcal carriage in all income classification were young age, ethnicity, symptoms of respiratory tract infection, childcare attendance, living with young children, poverty, exposure to smoke, season, and cocolonization with other pathogens. Breastfeeding and antibiotic use were protective against carriage in all income classifications. The relationship of carriage to the development of natural immunity remains poorly understood.

*S. pneumoniae* has a distinct polysaccharide capsule—a virulence factor allowing the bacteria to elude the immune defense mechanisms. Capsular polysaccharides are antigenic and form the basis for classifying pneumococci by the over 100 serotypes described to date. Type-specific antibody to capsular polysaccharide protects against the disease caused by that serotype. These antibodies interact with complement to opsonize pneumococci, facilitating phagocytosis and clearance of the organism. Antibodies to some pneumococcal capsular polysaccharides may crossreact with related types and with other bacteria, providing protection against additional serotypes [6].

Diagnosis of pneumococcal disease is based on Gram stain showing lancet-shaped diplococci, isolation of the organism from blood or other normal sterile body sites (eg, cerebrospinal fluid [CSF], middle ear fluid, joint fluid, and peritoneal fluid), or commercially available urinary antigen test based on an immunochromatographic membrane technique to detect the C-polysaccharide antigen of *S. pneumoniae*.

## 2. Clinical phenotypes of pneumococcal disease

Pneumococcal Disease in adults may present as pneumococcal pneumonia, pneumococcal bacteremia without pneumonia (eg, septic arthritis, meningitis, endocarditis with 12% overall case fatality ratio), or Pneumococcal meningitis (with 14% case fatality ratio).

Pneumococcal disease in children may present as pneumococcal pneumonia (accounts for 25%–30% of invasive disease in children aged ≤2 years), Pneumococcal bacteremia (accounts for 40% of invasive disease in children aged ≤2 years), and pneumococcal meningitis (leading cause of bacterial meningitis among children aged <5 years).

Conditions that increase the risk of IPD in children include chronic heart disease, lung disease (including asthma if treated with high-dose oral corticosteroid therapy), liver disease, CSF leak, having a cochlear implant, and functional or anatomic asplenia (eg, persons with sickle cell disease) [6]. Infection rates are also increased among children of certain racial and ethnic groups, including Alaska Natives, African Americans, and certain American Indian groups (Navajo and White Mountain Apache).

High-risk groups for IPD or severe nonbacteremic pneumonia include the following [6, 7]:

- Infants and children aged <2 years.
- Adults ≥65 years old.
- Persons with chronic medical conditions (eg, chronic cardiovascular, pulmonary, renal, liver disease; diabetes mellitus; alcohol abuse; smoking; malignancy including those aged 5–64 years).
- Immunocompromised states (eg, hypogammaglobulinemia, functional or anatomic asplenia due to sickle cell disease or splenectomy) including those aged 5–64 years.

## 3. Immunological differences between pneumococcal polysaccharide and conjugate vaccine

The first pneumococcal vaccine, a 14-valent unconjugated purified pneumococcal polysaccharide vaccine (PPSV), was licensed for use in the United States in 1977, followed by the 23-valent PPSV in 1983. The first pneumococcal conjugate vaccine (PCV) was licensed in the United States in 2000.

**Figure 1.**
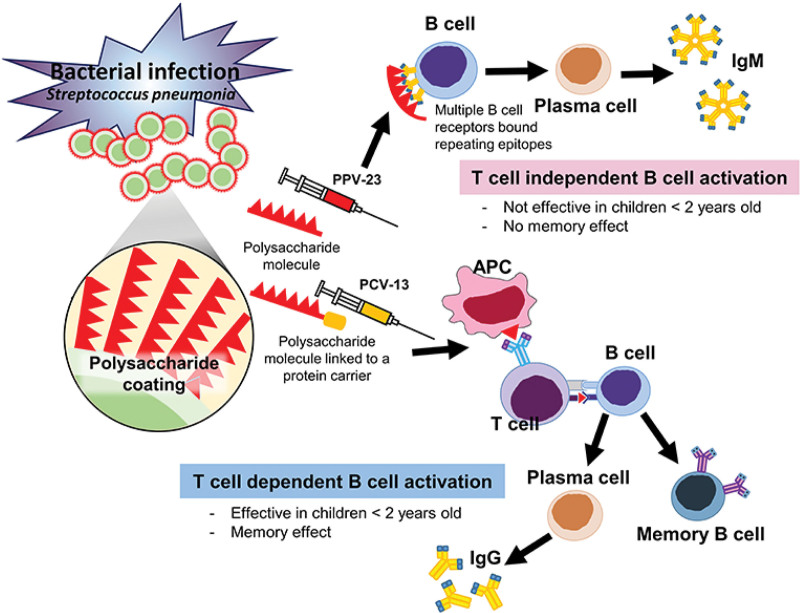
T-cell dependent B-cell activation (conjugated vaccine) vs T-cell independent B-cell activation (unconjugated polysaccharide vaccine) From Goonewardene et al. [8].

PPSV are T-cell independent and thus do not induce immunological B-cell memory. In contrast, conjugate vaccines are T-cell dependent antigens and induce immunological B-cell memory (Fig. 1) [8].

The conjugated formulation leads to a more robust and prolonged immune response. The first licensed 7-valent PCV used nontoxic variant of diphtheria toxin (CRM) as a carrier protein (PCV7-CRM). Subsequently, different proteins or protein complexes have been used as carriers in different formulations of pneumococcal vaccines in addition to CRM: diphtheria (DT) and tetanus toxoids, meningococcal outer membrane protein complex, and protein D (PD) of *Haemophilus influenzae*. Currently, commercially available PCV13 (Prevnar-13) and PCV20 (Prevnar-20) (Wyeth Pharmaceuticals) are conjugated with the Diphtheria CRM_197_ Protein [9].

Vaccine selection pressure leads to replacing the formerly dominant vaccine serotypes by nonvaccine types (NVTs). Serotyping is thus important for epidemiological surveillance and vaccine assessment, precisely monitor virulent lineages, NVT emergence, and genetic associations of isolates. Serotyping can be performed using numerous techniques, either by the conventional antisera-based (Quellung and latex agglutination), molecular-based approaches (sequetyping, multiplex polymerase chain reaction (PCR), real-time PCR, and PCR-restriction fragment length polymorphism), and potentially the whole genome sequencing to be directed for future exploration [9].

## 4. Pneumococcal vaccines and vaccination schedule

The currently registered and approved pneumococcal vaccines and their serotype coverage are as follows [1]:

- PCV13 (Prevnar-13): contains 13 serotypes of *S. pneumoniae* (1, 3, 4, 5, 6A, 6B, 7F, 9V, 14, 18C, 19A, 19F, and 23F) conjugated to a nontoxic variant of DT known as CRM197. PCV13 is administered by intramuscular injection. Each dose of PCV13 contains aluminum phosphate as an adjuvant. It contains no antibiotics or preservatives.
- PCV15 (Vaxneuvance): serotype coverage: 22F and 33F, in addition to PCV13 serotypes.
- PCV20 (Prevnar-20): serotype coverage: 8, 10A, 11A, 12F, 15B, 22F, and 33F, in addition to PCV13 serotypes.
- PPSV23 (Pneumovax23): serotype coverage: 1, 2, 3, 4, 5, 6B, 7F, 8, 9N, 9V, 10A, 11A, 12F, 14, 15B, 17F, 18C, 19A, 19F, 20, 22F, 23F, and 33F. It is administered by either intramuscular or subcutaneous injection. Each dose of PPSV23 contains phenol as a preservative. It contains no adjuvants or antibiotics.

There are PCV21, PCV23, and PCV25 conjugate vaccines undergoing phase I/II clinical trials currently not commercially available [1]. The summary of the vaccination schedules in children and adults are as follows [10–13]:

- PCV13:
  - Three-dose primary series at age 2, 4, and 6 months.
  - Booster at age 12 through 15 months.
  - Minimum age for dose 1 is 6 weeks.
  - Minimum interval for doses before age 1 year is 4 weeks and age ≥1 year is 8 weeks.
  - Unvaccinated children aged 7 months or older require fewer doses.
  - Shared clinical decision-making for adults aged ≥65 years.
- PPSV23:
  - One dose for all adults aged ≥65 years.
- Schedule for PCV13 and PPSV23 varies by medical condition (see Table 1).

**Table 1.** Conditions with pneumococcal vaccination indications

Conditions	PCV13 indicated for 2–71 months*	PCV13 indicated for 6–18 years*	PCV13 indicated for 19 years or older	PPSV23 indicated for 2–64 years
Chronic heart or lung disease†	**√**	–	–	**√**
Diabetes	**√**	–	–	**√**
Chronic liver disease, including cirrhosis	**√**	–	–	**√**
Cigarette smoking (in adults)	**√**	–	–	**√**
Alcoholism (in adults)	**√**	–	–	**√**
Cerebrospinal fluid leak	**√**	**√**	**√**	**√**
Cochlear implant	**√**	**√**	**√**	**√**
Functional or anatomic asplenia, including sickle cell disease	**√**	**√**	**√**	**√**
Immunocompromising conditions‡	**√**	**√**	**√**	**√**
Persons living in special environments or social settings§	–	–	–	Consider

PCV indicates pneumococcal conjugate vaccines; PPSV, purified pneumococcal polysaccharide vaccine.

* PCV13 only recommended if child is unvaccinated or received incomplete vaccine schedule.

† Includes congestive heart failure, cardiomyopathies, chronic obstructive pulmonary disease, emphysema, and asthma. Asthma only included for children (through age 18 years) if treated with high-dose oral corticosteroid therapy.

‡ Includes congenital or acquired immunodeficiencies, Hodgkin’s Disease, lymphoma, leukaemia, multiple myeloma, generalized malignancy, and other cancers if on immunosuppressive therapy; HIV infection; chronic renal failure; nephrotic syndrome; organ transplant; and immunosuppressive medications, including chemotherapy and high-dose corticosteroid treatment.

§ Includes Alaska Native, Navajo, and White Mountain Apache populations.

Adapted from Gierke R, et al. The Pink Book: Course textbook. 14th ed. 2021.

The summary of the vaccination approach for selected chronic medical conditions is as follows [10–14]:

- Chronic heart disease, chronic lung disease, diabetes, alcoholism, chronic liver disease including cirrhosis, current cigarette smoking, and asthma:
  - 19–64 years: single dose PPSV23
  - ≥65 years:
    - PCV13 (shared decision-making).
    - One dose of PCV13 then 1 dose of PPSV23 after 5 years.
- Cerebrospinal fluid leak, cochlear implant:
  - 19–64 years: 1 dose of PCV13 followed by 1 dose of PPSV23 after 8 weeks.
  - ≥65 years: 1 dose PCV13 followed by 1 dose of PPSV23 after 8 weeks followed by 2nd dose PPSV23 after 5 years (if any PPSV23 given before 65 years).
- Functional and anatomic asplenia (including sickle cell disease/other hemoglobinopathies) and immunocompromising conditions [14]:
  - 19–64 years: 1 dose of PCV13 followed by 1 dose of PPSV23 after 8 weeks followed by 2nd dose of PPSV23 after 5 years (if any PPSV23 given before 65 years).
  - ≥65 years: 1 dose PCV13 followed by 1 dose of PPSV23 after 8 weeks followed by 2nd dose PPSV23 after 5 years (if any PPSV23 given before 65 years).

PCV20 (Prevnar-20), the newest conjugated vaccine commercially available in 2023 covers serotype 8, 10A, 11A, 12F, 15B, 22F, and 33F, in addition to PCV13 serotypes. A 4-dose childhood immunization series at 2, 4, 6, and 12 to 15 months of age; and a single dose in adults aged ≥18 years has been shown to be effective in the prevention of the following [15–17]:

- Invasive disease caused by all 20 *S. pneumoniae* serotypes in individuals aged ≥6 weeks.
- Pneumonia caused by all 20 *S. pneumoniae* serotypes in individuals aged ≥18 years.
- Otitis media caused by only 7/20 *S. pneumoniae* serotypes 4, 6B, 9V, 14, 18C, 19F, and 23F in individuals aged 6 weeks to 5 years.

Mortality from IPD has improved dramatically as a result of improved vaccination rates through national childhood and adult immunization programs, and improved serotype coverage with existing vaccines in particular PCV13 and PPSV23. Serial vaccination with both conjugate and purified polysaccharide vaccines has produced greater and longer-lasting immunity. The development of the conjugated PCV15 [13] and the PCV20 [15] vaccine in adults allow vaccine-mediated immunity against a wider spectrum of pneumococcal serotypes not covered by PCV13. Since the introduction of PCV13, invasive disease caused by PCV13 serotypes declined 90% in children.

## 5. Evaluating pneumococcal vaccine effectiveness

Immunity to pneumococcal infection may result from immunity acquired through natural infection or in response to PPSV or PCV vaccination. Assessment of vaccine effectiveness comprises quantitative and qualitative measurements of function. Laboratory measurement of immunity comprises the following:

- Quantitation of antibodies—radioimmunoassays, enzyme immunoassays, Luminex method
- Functional activity of antibodies—opsonophagocytosis assay
- Assessment of antibody avidity
- Assessment of cell-mediated immunity
- Assessment of immunological memory—direct measurement and characterization of memory B-cells after booster immunization using flow cytometric or enzyme-linked immunospot assay techniques

Immunological memory to a polysaccharide antigen is defined as a response that is present in otherwise nonresponsive individuals (eg, infants), characterized by a higher antibody response with IgG dominance on exposure to an antigen, and dominated by antibodies with increased avidity. In the real-world clinic setting, laboratory quantitation of pre- and 4–6 week post-PPSV vaccination antibody levels by serotype is usually carried out in children and adults with suspected inborn errors of immunity (IEI) [18–20] or secondary hypogammaglobulinemia [21]. Causes of impaired polysaccharide responsiveness include the following:

- Common variable immunodeficiency [22]
- Selective antibody deficiency: in the presence of normal or increased immunoglobulin levels, including IgG subclasses, and intact humoral response to protein antigens [23, 24]; or B lymphocyte defects [25]
- Other IEI: including Wiskott–Aldrich syndrome, autoimmune lymphoproliferative syndrome, and DiGeorge syndrome, IgG-subclass deficiencies.

Antibody concentrations greater than or equal to the reference value for at least 50% of serotypes in either a pre- or postvaccination specimen or a 2-fold or greater increase in antibody concentrations for at least 50% of serotypes when comparing the pre- to the postvaccination results is usually taken as a normal response to *S pneumoniae* vaccination. Serotype-specific antibodies may persist for up to 10 years following immunization or infection. Limitations of some of these commercially available assays include database of IgG antibody concentrations to different serotypes being incomplete, unknown protective concentrations of IgG antibodies, or those required to prevent infection from *S. pneumoniae* and quantitation of the IgG antibody response to pneumococcal serotypes not providing any information on the functional capacity of the serotype- specific antibodies generated (opsonization efficiency).

## 6. Pneumococcal vaccine adverse reactions

Pneumococcal vaccination is contraindicated where there has been a previous allergic reaction following any type of pneumococcal conjugate vaccine (PCV13, PCV15, PCV20, or an earlier pneumococcal conjugate vaccine known as PCV7) or PPSV23, or to any vaccine containing DT (eg, DTaP) as the latter is a PCV vaccine component. Adverse reactions to vaccines are not uncommon including injection site pain, swelling, erythema, fever, loss of appetite, fatigue, headache, muscle aches, joint pain, and chills. Allergic reactions to vaccines [26], in particular anaphylaxis [27], are generally rare. Case reports of immediate [28–30] and delayed [31, 32] allergic reactions to pneumococcal vaccines have been published, although overall these reactions are rare [33]. When immediate hypersensitivity reactions occur, diagnostic allergological evaluation should include skin testing vaccine components including excipients (where possible). In the absence of positive skin tests and a history consistent with an immediate reaction, other non-IgE mediated mechanisms for vaccine hypersensitivity for example complement-activated related pseudoallergy should be considered [34, 35].

## 7. Conclusion

With the expanding pipeline of PCV vaccines with improved immunogenicity to reduce the morbidity and mortality associated with pneumococcal disease across all ages, vaccine-resistant serotypes may paradoxically emerge. The increasing diversity, heterogeneity, and complexity of IEI in children and adults makes it imperative for clinical immunologists/allergists to continue to deepen their understanding of the immunology surrounding pneumococcal vaccines. Both PPSV and PCV continue to play major roles in the diagnosis of IEI and the prevention of pneumococcal disease in patients at high risk of IPD.

## Conflicts of interest

The authors have no financial conflicts of interest.

## Author contributions

Conceptualization and writing: Bernard Yu-Hor Thong and Ruby Pawankar. Review and feedback: Hae-Sim Park and Amir Hamzah Abdul Latiff.
